# Geographic distribution of need and access to health care in rural population: an ecological study in Iran

**DOI:** 10.1186/1475-9276-10-39

**Published:** 2011-09-22

**Authors:** Aliasghar Ahmad Kiadaliri, Behzad Najafi, Hassan Haghparast-Bidgoli

**Affiliations:** 1 Division of Health Economics, Department of Clinical Sciences, Malmö; Skåne University Hospital; Lund University, MALMÖ SE-20502, Sweden; 2 Health Economics & Management, Institute of Economic Research, Lund University, Lund, Sweden; 3 Department of Health Management and Economics, School of Public Health, Tehran University of Medical Sciences, Tehran, Iran; 4 Department of Health Care Management, School of Management and Medical Informatics, Tehran University of Medical Sciences, Tehran, Iran; 5 School of Public Health, Ardebil University of Medical Sciences, Ardebil, Iran; 6 Department of Public Health Sciences, Division of Global Health, Karolinska Institute, Stockholm, Sweden

**Keywords:** Equality, Gini Index, Index of Dissimilarity, Rural Health Houses, Iran

## Abstract

**Introduction:**

Equity in access to and utilization of health services is a common goal of policy-makers in most countries. The current study aimed to evaluate the distribution of need and access to health care services among Iran's rural population between 2006 and 2009.

**Methods:**

Census data on population's characteristics in each province were obtained from the Statistical Centre of Iran and National Organization for civil registration. Data about the Rural Health Houses (RHHs) were obtained from the Ministry of Health. The Health Houses-to-rural population ratio (RHP), crude birth rate (CBR) and crude mortality rate (CMR) in rural population were calculated in order to compare their distribution among the provinces. Lorenz curves of RHHs, CMR and CBR were plotted and their decile ratio, Gini Index and Index of Dissimilarity were calculated. Moreover, Spearman rank-order correlation was used to examine the relation between RHHs and CMR and CBR.

**Results:**

There were substantial differences in RHHs, CMR and CBR across the provinces. CMR and CBR experienced changes toward more equal distributions between 2006 and 2009, while inverse trend was seen for RHHs. Excluding three provinces with markedly changes in data between 2006 and 2009 as outliers, did not change observed trends. Moreover; there was a significant positive relationship between CMR and RHP in 2009 and a significant negative association between CBR and RHP in 2006 and 2009. When three provinces with outliers were excluded, these significant associations were disappeared.

**Conclusion:**

Results showed that there were significant variations in the distribution of RHHs, CMR and CBR across the country. Moreover, the distribution of RHHs did not reflect the needs for health care in terms of CMR and CBR in the study period.

## Introduction

Following Alma-Ata declaration on the key role of primary health care (PHC) in achieving health for all and decreasing inequality in health [1], the Iranian government attempted to develop an extensive network of PHC facilities, especially in rural areas. PHC in Iran's rural areas are mainly provided through the rural health houses (RHHs). RHHs, which are considered as the main component of progressive expansion of PHC coverage, are aimed at reducing the urban-rural gap in Iran's health care delivery system [2]. Following a series of pilot projects in early of 1970s, RHHs were introduced in 1981 [3].

**Figure 1:**
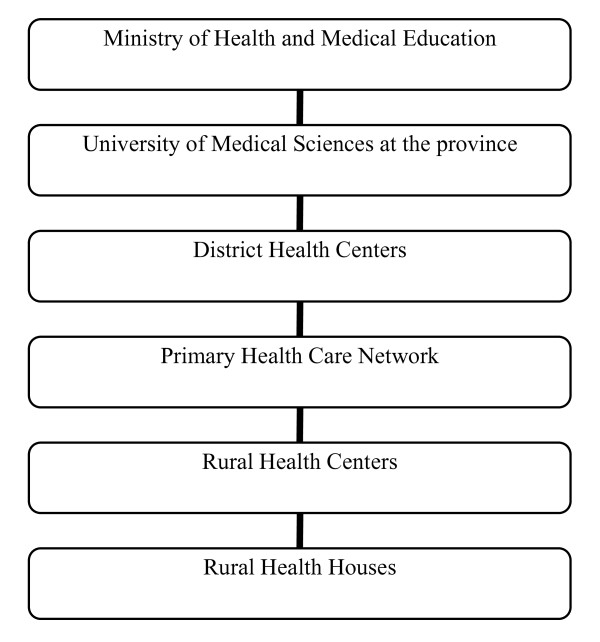
**Position of Rural Health Houses (RHHs) in the health care delivery system of Iran**.

RHHs act as the first level of contact to the basic PHC in Iran's rural areas. These units serve a population of 1,500 people who are living in the main village (where RHH is located), and satellite villages (which are an hour walk distance from the main village). Two trained local residents, who are known as Behvarz (one male and one female), work as health workers in each RHH and provide PHC services including maternal and child health care, family planning, vaccinations and environmental health promotion to the rural population. Moreover, they are responsible for referring patients who need further care to the next level of care. Figure 1 shows the position of RHHs in the health care delivery system of Iran.

During past decades, the number of rural health houses has continuously increased in the country. However, it is not just the quantity of health care resources which affect health status of people, but how they are distributed is also important. Inequitable distribution of health services is a major barrier for improving health service delivery for health systems around the world [4]. There is indeed a positive linkage between availability of health care resources and health status of population [5]. For this reason, the distribution of health care is considered as one of the social determinants of health [6].

In a sense, the geographic distribution of health facilities is considered as a major health policy issue in many countries, both developed and developing. It is believed that the utilization of, and access to, healthcare among individuals should not be affected by the geographical region in which they reside [7].

Although studies in Iran have examined the effectiveness of rural health houses in improvement of the population's health status [8] and decreasing the disparities between rural and urban areas [9], little attention has been paid to the distribution of RHHs within rural areas in the country.

The current study examines availability of RHHs in Iran using inequality measures. This study specifically focuses on the following research questions: How were RHHs, crude mortality rate (CMR) and crude birth rate (CBR) distributed between different provinces in years 2006 and 2009? How has changed the inequality measures between 2006 and 2009? Did the distribution of RHHs reflect CMR and CBR in the rural population of the provinces?

## Material and methods

### Study setting

Iran, a lower-middle-income country, is located in Eastern Mediterranean Region with an area of 1,648,000 km sq. It has a population of about 73.5 million people; of whom about 23 million are living in rural areas [10]. The country has 30 provinces, 293 districts, 885 cities and more than 68,000 villages [11]. Table 1 shows some of the main health indicators for the country [10].

**Table 1:** Some main health indicators in Iran

Indicator	Value	Year ^a^
Crude birth rate (per 1000 people)	18.3	2006
Crude mortality rate (per 1000 people)	5.72	2009
Population with access to improved sanitation (%)	71	2008
Total expenditure on health of % of GDP	6.3	2008
Out-of-pocket expenditure as % of total health expenditure	51.7	2008
Physicians per 10,000 population	8.9	2008
primary health care units and centres per 10,000 population	3.1	2006
Population with access to local health services, total (%)	99	2009
Total life expectancy at birth (years)	72.1	2006
Infant mortality rate (per 1000 live births)	27	2005
Under five mortality rate (per 1000 live births)	27.7	2005
Maternal mortality ratio (per 10000 live births)	25	2005

a Reference year for data provided

### Data sources and variables

The census and estimated data on the distribution and characteristics of population at province level were obtained from the Statistical Centre of Iran [12]. The data about the number of total births and mortalities were also obtained from National Organization for Civil Registration [13]. The data on the number of RHHs in the provinces were gathered from the Statistics Centre of Ministry of Health and Medical Education (MOHME). The centre collects data about health facilities and other health indicators from the Medical Universities in the provinces.

In the current study, the number of RHHs per 1000 rural people (RHP) was used as the indicator for availability of health care resources for the rural population in each province. Moreover, two variables including CMR (number of deaths per 1000 rural people) and CBR (number of births per 1000 rural people) were selected to show the community's health needs. CMR have been used in the literature as an estimate of community health need [14-16]. In addition, CBR was used as another community health need indicator in this study since health services for newborns and infants are one of the main components of the services provided by RHH.

To see how the distribution of access and need to health care services has changed over time, the data for years 2006 and 2009 (as latest available data) were gathered. Moreover, this enables us to control for potential measurement bias in the data as each year can used as a control for other year.

### Inequality Indicators

#### Lorenz Curve and Gini index

The Gini index and Lorenz curve are commonly used in analyzing the inequality in distribution of health care resources [17-19]. Lorenz curve is used to compare distribution of specific health variable with perfect equality (diagonal line). This curve plots the cumulative share of population ranked by health variable, in an increasing order, against the cumulative share of health variable. The further the distance from diagonal line, the greater the degree of inequality. The area between the Lorenz curve and diagonal line present a measure of inequality entitled Gini Index. The Gini index is equal to twice the area between the Lorenz curve and diagonal. The magnitude of the Gini index ranges from 0 (perfect equality) to one (maximum possible inequality). In the current study, the formula proposed by Brown [20] was used for calculating the Gini index as follow:

$$G=\text{ 1-}\sum_{\text{i}=\text{0}}^{\text{k-1}}\left(Y_{i+1}+{\text{Y}}_{\text{i}}){\text{ (X}}_{\text{i}+\text{1}}{\text{ - X}}_{\text{i}}\right)$$

G: Gini Index

Y_i_: cumulative share of RHH in the ith province

X_i_: cumulative share of population (ranked by RHP) in the ith province

k: total number of provinces

### Decile ratio

To calculate the decile ratio, the provinces were ranked by RHP. The top 10% from the top ratio is then divided by the 10% of the bottom.

### Index of Dissimilarity

This index estimates the proportion of total health variable which would need to be transferred from provinces with health variable values higher than the country's mean to those which values lower than the country's mean to achieve a situation of perfect equality [21]. It is calculated through the formula:

$$ID\;=\;\frac{\text{1}}{\text{2}}\;\overset{\text{n}}{\underset{\text{i}=\text{1}}{\sum }}|p_{ip}-{\text{p}}_{\text{ih}}|$$

p_ip_: ith province's population share

p_ih_: ith province's health variable share

### Data analysis

In the current study, the geographic unit of analysis is 30 provinces in Iran. RHP for each province was calculated as the number of RHHs per 1000 rural people. This ratio was used to rank the provinces in drawing the Lorenz curve and calculating the Gini index and decile ratio for access indicator. In case of need indicators, the CBR and CMR were used to rank the provinces.

Inequality measures (including Lorenz curve, Gini, decile ration and dissimilarity index) were used to assess the level of inequality in the distribution of RHHs, CMR and CBR across the provinces.

The spearman rank-order correlation coefficients between RHP, CMR and CBR were calculated to examine if there is any linear relationship between distribution of RHHs and community health needs. Correlation measures have been used in some other studies to evaluate the linear relationship between two variables in examining the inequality in health [19,22].

To find the potential outliers in data, the box plots of percentage changes in CBR and CMR between 2006 and 2009 were used. The results are reported for total sample and non-outlier sample where outliers were removed from total sample. Moreover, in order to explore changes in CMR and CBR over study period; the median was used due to these outliers in the data.

## Results

Table 2 shows the distribution of RHP, CMR and CBR in the provinces of Iran for years 2006 and 2009. There were substantial differences in all indicators across the country. The national mean value of RHP increased by 7% from 0.75 in 2006 to 0.80 in 2009. Of the 30 provinces, Tehran was the province with the lowest RHP in both 2006 and 2009, although its RHP increased from 0.26 in 2006 to 0.31 in 2009. Yazd had the highest RHP in both years, with 1.05 and 1.14 in 2006 and 2009, respectively. The differences between the provinces with the highest and the lowest RHP have reduced (from 4.04-fold in 2006 to 3.70-fold in 2009) over the study period. Except for Fars province, the RHPs increased in all provinces between 2006 and 2009. Among the provinces, Qazvin had the highest increase in RHP with 22% and Sistan & Baluchestan had the lowest increase with 1%.

**Table 2:** Population ^a^, RHP ^b^, CMR ^c ^and CBR ^d ^in provinces of Iran, 2006 and 2009

Province	2006				2009			
	
	Population	RHP	CMR	CBR	Population	RHP	CMR	CBR
Ardebil	512.558	1.01 (5)^e^	5.19	16.13	473.449	1.09 (6)	5.81	18.16
Bushehr	308.802	0.67 (24)	6.83	18.85	280.982	0.77 (21)	4.85	21.47
Chaharmahal	415.612	0.66 (25)	5.70	21.98	407.188	0.74 (23)	4.82	23.32
East Azarbaijan	1200.917	0.88 (10)	5.94	18.62	1137.522	0.94 (13)	7.70	20.75
Fars	1683.931	0.61 (28)	5.93	15.75	1664.832	0.56 (29)	5.94	16.26
Gilan	1109.110	0.85 (14)	6.90	10.92	1063.204	0.91 (15)	10.00	11.84
Golestan	821.961	0.71 (22)	4.91	22.70	799.663	0.75 (22)	5.48	25.19
Hamedan	722.496	0.79 (18)	5.93	17.76	663.813	0.86 (19)	5.97	20.11
Hormozgan	742.349	0.63 (27)	28.91	23.00	773.469	0.71 (26)	4.16	22.55
Ilam	214.556	0.88 (12)	5.09	18.33	205.286	0.95 (10)	5.05	16.81
Isfahan	760.528	0.85 (15)	6.87	12.81	634.372	0.95 (12)	6.05	16.31
Kerman	1099.894	0.69 (23)	3.23	17.94	1130.619	0.73 (25)	3.30	19.46
Kermanshah	624.066	1.04 (2)	5.36	15.91	595.599	1.12 (3)	4.72	15.29
Khuzestan	1401.415	0.60 (29)	11.11	21.60	1372.389	0.63 (28)	3.49	21.81
Kohkiluyeh	332.107	0.94 (7)	13.54	21.35	324.817	1.00 (8)	7.79	21.77
Kordestan	584.337	1.03 (3)	5.16	18.25	555.509	1.11 (5)	5.58	18.47
Lorestan	696.377	0.88 (11)	5.98	19.41	672.260	0.93 (14)	4.69	21.17
Markazi	419.184	0.98 (6)	6.78	13.12	367.709	1.12 (4)	9.27	14.23
Mazandaran	1368.289	0.87 (13)	6.03	13.42	1327.959	0.89 (16)	8.90	14.34
North Khorasan	419.114	0.80 (17)	5.48	22.93	403.909	0.87 (17)	6.70	23.69
Qazvin	365.225	0.71 (21)	5.75	16.33	332.871	0.87 (18)	7.52	16.07
Qom	63.643	0.93 (8)	3.66	9.87	54.347	1.07 (7)	4.23	12.02
Razavi Khorasan	1781.179	0.71 (20)	5.08	18.93	1711.904	0.73 (24)	7.07	19.78
Semnan	149.183	0.92 (9)	5.80	13.02	141.201	0.95 (11)	13.05	13.41
Sistan & Baluchestan	1212.544	0.65 (26)	8.80	34.21	1300.819	0.65 (27)	5.82	30.71
South Khorasan	309.725	0.81 (16)	7.11	19.00	297.735	0.95 (9)	21.27	18.21
Tehran	1161.935	0.26 (30)	1.74	9.57	962.542	0.31 (30)	2.66	9.36
West Azarbaijan	1148.505	0.76 (19)	4.67	23.65	1110.783	0.85 (20)	5.09	23.98
Yazd	201.015	1.05 (1)	4.53	10.03	185.569	1.14 (1)	5.60	12.66
Zanjan	405.261	1.01 (4)	5.59	19.32	373.461	1.13 (2)	7.25	19.36
Median	-	0.83	5.78	18.29	-	0.90	5.82	18.92
Mean	-	0.75	6.80	18.39	-	0.80	6.21	19.27

a In thousands

b Rural health houses per 1000 rural people

c Crude Mortality Rate (per 1000 rural people)

d Crude Birth Rate (per 1000 rural people)

e Numbers in parentheses show the provinces ranks.

The median of CMR increased 0.7% from 5.78 in 2006 to 5.82 in 2009. Tehran had the lowest CMR in both 2006 and 2009. Hormozgan and South Khorasan were the provinces with the highest CMR in 2006 and 2009, respectively. There were 16.5- and 8-fold differences between provinces with the highest and the lowest CMR in 2006 and 2009, respectively. 20 out of 30 provinces experienced an increase in CMR between 2006 and 2009.

The median of CBR increased 0.4% over the study period. Tehran had the lowest CBR in both 2006 and 2009. The highest CBR was seen in Sistan & Baluchestan in the same period. There were 3.6- and 3.3-fold differences between the provinces with the highest and the lowest CBR in 2006 and 2009, respectively. 23 out of 30 provinces experienced an increase in CBR between 2006 and 2009.

As provinces of Hormozgan, Semnan and South Khorasan had the outlier values on variables, we reported the total sample results and results after excluding these provinces separately. As there were no significant changes in general results and interpretation, the results of total sample will be discussed in following sections.

**Figure 2:**
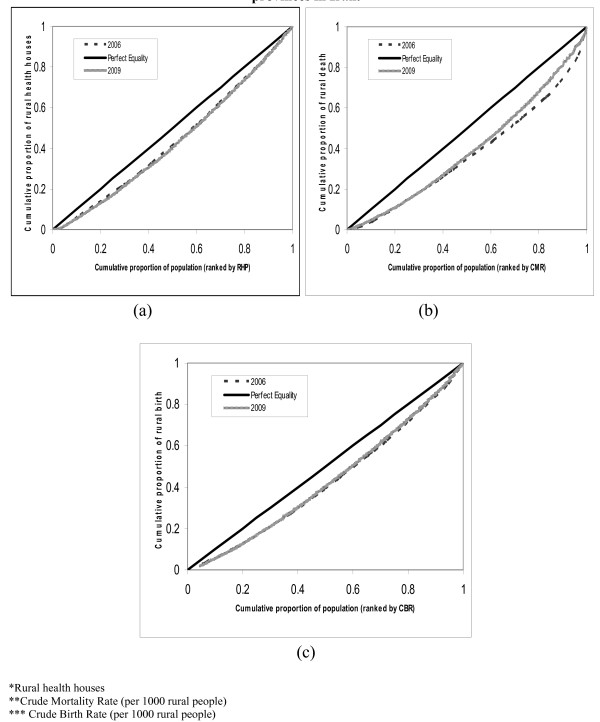
**Lorenz curves of distribution of: (a) RHHs, (b) CMR and (c) CBR among provinces in Iran**.

Overall distribution of access and need indicators across the country in 2006 and 2009 are shown in Figure 2. A comparison between Lorenz curves showed that there are no evident changes in the distribution of RHH and the CBR between 2006 and 2009 (Figure 2). However, the distribution of CMR approached to the perfect equality line.

Table 3 shows the inequality indicators for RHH, CMR and CBR in 2006 and 2009. Among the variables, RHHs were most equally distributed and the distribution of CMR was least equal. The Gini coefficient for RHH increased by 6.4% between 2006 and 2009, while during the same period the Gini for CBR and CMR decreased by 10.8% and 20.8%, respectively.

**Table 3:** Inequality indicators of the distribution of RHHs, Deaths and Births in rural area of Iran

Variable	Total sample					
	
	2006			2009		
	
	Gini coefficient	Index of Dissimilarity	Decile ratio	Gini coefficient	Index of Dissimilarity	Decile ratio
RHH^a^	0.125	9% (1505)	2.38	0.133	10% (1670)	2.58
Death	0.260	18% (27335)	6.70	0.206	14% (18887)	3.70
Birth	0.158	11% (43732)	2.58	0.141	10% (40338)	2.61

	Outliers excluding sample ^b^					
	
RHH^a^	0.126	9% (1435)	2.59	0.135	10% (1602)	2.58
Death	0.185	12% (15189)	4.75	0.177	12% (14815)	3.19
Birth	0.162	11% (42991)	2.96	0.143	10% (38860)	2.70

a Rural health house

b Three provinces (Hormozgan, Semnan and South Khorasan) were excluded as outliers.

Table 4 shows the results of the correlation analyses between the access indicator (RHP) and the need indicators (CMR and CBR) in 2006 (Table 3). There was a moderate direct statistically significant relationship between CMR and RHP in 2009. On the other hand, the relationship between CBR and RHP was moderately negative in 2006 and 2009; implying that people with higher health needs had lower access to RHHs. When we excluded the provinces with outliers, there were no significant associations between access and need indicators.

**Table 4:** Correlation between RHP, CMR and CBR in Iran in 2006 and 2009

Variable	Total sample	
	
	2006	2009
	RHP ^a^	RHP
	
CMR ^b^	- 0.15 (0.42) ^d^	**0.37 **(0.04)
CBR ^c^	- 0.34 (0.06)	**- 0.41 **(0.03)

	Outliers excluding sample ^e^	
	
CMR	- 0.06 (0.77)	0.31 (0.12)
CBR	- 0.24 (0.24)	- 0.31 (0.11)

a Rural health houses per 1000 rural people

b Crude Mortality Rate (per 1000 rural people)

c Crude Birth Rate (per 1000 rural people)

d Spearman rank order correlation with p values in parentheses.

e Three provinces (Hormozgan, Semnan and South Khorasan) were excluded as outliers.

## Discussion

The current study assessed access to and need for health services in rural areas of Iran for the years 2006 and 2009. The study showed that the distribution of RHHs is not based on need in terms of CMR and CBR across the provinces in Iran. Moreover, the results indicated significant regional variations in both access and need indicators across the country. To achieve an equal distribution of RHHs across the country, about one out of 10 RHHs should be re-allocated from the relatively over-served provinces to the relatively under-served ones.

The results showed that RHHs are not distributed based on CMR and CBR in the provinces. One possible explanation for these results is maybe that policy makers consider some other indicators than those used in this study (CMR and CBR) for the distribution of RHHs across the country (for example; having a minimum number of RHHs for each province or socio-economic situation of the provinces).

Although, access to RHHs (measured by RHP) improved between 2006 and 2009, the needs for health services (measured by CMR and CBR) increased at the same time. In terms of inequality measures, there were changes in need indicators toward more equal distributions, while inverse trend was seen for the access indicator.

The degree of inequality in the distribution of RHH and the CBR were rather stable during the study period, while it significantly changed for CMR in total sample. However, when we excluded the outliers, then no significant changes in inequality indicators were observed. Generally, there was no strong association between the distribution of RHH and CMR and CBR in rural areas of Iran.

Previous studies mostly evaluated the rural-urban differences in distribution of health care resources and outcomes, not differences within the rural areas [14]. Among the few studies within rural areas, Theodorakis et al [23] reported an uneven distribution of primary care physicians in remote areas of Greece and Albania. Another study in the USA in 2005 indicated unequal distribution of physician among rural areas [24].

The results of this study however should be interpreted in light of some limitations. Firstly, the data are gathered from census data which are subject to incompleteness and measurement errors and these may bias the results. For example, undercounting, misreporting and delayed registration are some well known problems of census and mortality data in Iran [25]. These possibly explain the outliners in our data. Secondly, the data used in the study are aggregated data at the province level. It implies that the variation within the provinces could be higher than the variation between the provinces. Hence, these results are not necessarily applicable to smaller geographic units such as counties or cities. Thirdly, need variables used in this study were crude measures and sex and age differences in these measures were not taken into account due to the lack of data. Fourthly, our results are limited to geographical comparisons; without knowing who actually use the services provided by RHHs, one cannot know their distribution according to other dimensions of the population, such as income, education and etc.

## Conclusion

This study showed that the distribution of RHHs does not reflect the needs for health care in terms of CMR and CBR. There were significant variations in the distribution of RHHs, CMR and CBR across the country. While the inequality in access increased during the study period, the inequality in need for health care decreased at the same time in the rural areas. It is suggested that the results of this study be considered in making decisions on rural health care services by policy-makers in Iran.

## Competing interests

The authors declare that they have no competing interests.

## Authors' contributions

AAK participated in design of study, analysis and interpretation and drafting the manuscript. BN participated in acquisition the data, acquisition of funding and revision of the manuscript. HHB participated in interpretation of results and revision of the manuscript. All authors read and approved the final manuscript.
